# The Commonwealth of Cells

**Published:** 1902-06

**Authors:** 


**The Commonwealth of Cells.** By H. G. F. SPURRELL. Pp. xi., 115. London: Baillière, Tindall & Cox. 1901.

We are often asked to recommend a small book on physiology that gives a simple and readable outline, and which can be understood by those who have had no training in the sciences. This book will answer the purpose admirably.

As the title indicates, the author keeps before us throughout the book the cellular structure of the various tissues of the body, with the result that a more uniform and connected survey is obtained than if he had followed the more usual text-book methods. There is here no need to wade through pages of "dry-as-dust" details of formulæ, theories and facts that are only of value to the student.

The book is not free from error, and the illustrations and text are at times too clumsy to be lucid; but we hope that a further edition will be called for, as the book is thoroughly well worth re-writing.

